# The Anti-Aggregative Peptide KLVFF Mimics Aβ1-40 in the Modulation of Nicotinic Receptors: Implications for Peptide-Based Therapy

**DOI:** 10.3390/biomedicines10092231

**Published:** 2022-09-08

**Authors:** Hanna Trebesova, Guendalina Olivero, Mario Marchi, Massimo Grilli

**Affiliations:** Department of Pharmacy, Section of Pharmacology and Toxicology, University of Genoa, 16148 Genoa, Italy

**Keywords:** KLVFF, β-amyloid, synaptosomes, Alzheimer’s disease, nicotinic receptors, muscarinic receptors, dopamine release, noradrenaline release

## Abstract

In recent years, the inhibition of beta-amyloid (Aβ) aggregation has emerged as a potential strategy for Alzheimer’s disease. KLVFF, a small peptide corresponding to the aminoacidic sequence 16-20 of Aβ, reduces Aβ fibrillation dose dependently. Therefore, the toxic and functional characterization of its brain activity is fundamental for clarifying its potential therapeutic role. Accordingly, we studied the modulatory role of KLVFF on the cholinergic receptors regulating dopamine and noradrenaline release in rat synaptosomes. Nicotinic receptors on dopaminergic nerve terminals in the nucleus acccumbens are inhibited by KLVFF, which closely resembles full-length Aβ1-40. Moreover, KLVFF entrapped in synaptosomes does not modify the nicotinic receptor’s function, suggesting that external binding to the receptor is required for its activity. The cholinergic agent desformylflustrabromine counteracts the KLVFF effect. Remarkably, muscarinic receptors on dopaminergic terminals and nicotinic receptors regulating noradrenaline release in the hippocampus are completely insensitive to KLVFF. Based on our findings, KLVFF mimics Aβ1-40 as a negative modulator of specific nicotinic receptor subtypes affecting dopamine transmission in the rat brain. Therefore, new pharmacological strategies using the anti-aggregative properties of KLVFF need to be evaluated for potential interference with nicotinic receptor-mediated transmission.

## 1. Introduction

Alzheimer’s disease (AD) remains a sword of Damocles hanging over our heads. The lack of significant improvements has blocked private investment in this area of the health sector [1]. In recent decades, we have witnessed the devaluation and revaluation of the main theories related to AD. The “amyloid hypothesis” is probably the most important and it has characterized the last several decades of AD research [2]. To date, clinical trials targeting beta-amyloid (Aβ) have largely failed their primary endpoints. In June 2021, the Food and Drug Administration (FDA) approved Aducanumab, which revived the amyloid cascade theory but left serious doubts [3,4]. This new drug is a monoclonal antibody selective for aggregated forms of Aβ. The scientific community has been divided because the supporting data are limited in terms of biomarker characterization and clinical efficacy. However, preclinical studies repeatedly provide evidence for the validity of the Aβ cascade in AD progression. Different reasons can explain the discrepancies between preclinical and clinical results. Aβ exists in different forms and can interact with many targets in the brain [2,5,6]. Therefore, it is difficult to counteract all the pathways involved. Moreover, the protein has a hormetic profile that oscillates between physiological and pathological effects that are not yet fully clarified [7]. Accordingly, the idea of blocking enzymatic production has not produced convincing results [8]. The need to protect patients from physiological effects has prompted researchers to investigate the reasons for pathological accumulation and aggregation [9,10]. It is known that aggregation is caused by multiple triggers and is often induced by a partially unfolded protein, resulting in the formation of an intramolecular interaction [11,12]. Given these studies, many researchers agree that one of the promising therapeutic approaches is aimed at modulating Aβ aggregation [13,14]. A recent paper summarizes the potential therapeutics able to interfere with amyloid’s aggregation [15]. The authors describe peptides as the most promising tools among metal chelators, nanostructures, organic molecules, peptide mimics, and antibodies. The literature consistently shows that a short peptide (KLVFF), derived from the cleavage of Aβ, can interfere with amyloid aggregation [16,17,18]. Considering the possibility of using this peptide therapeutically, we need to define the pharmacological and toxicological profile and highlight the potential targets involved. The monomeric form of Aβ interferes with different synaptic processes such as exocytosis, endocytosis, and trafficking. It is plausible that some of these processes are sequence-selective and that therefore they can be mimicked by specific fragments. Interestingly, we demonstrated in a previous publication that Aβ modulated cholinergic receptors, both muscarinic and nicotinic subtypes, and bound differently in or out active sites [19]. This modulation differently affects dopamine and noradrenaline neurotransmission in rat brain, describing a part of the neurotransmitter homeostasis affected by the full-length peptide [20,21,22]. Consequently, in the present work, we decided to study the impact of KLVFF on synaptic integrity and cholinergic receptors regulating neurotransmitter release in rat brain synaptosomes.

## 2. Materials and Methods

### 2.1. Animals and Brain Tissue Preparation

Adult male rats (Sprague–Dawley, 200–250 g) were housed at constant temperature (22 ± 1 °C) and relative humidity (50%) under a regular light–dark schedule (light 7.00 a.m.–7.00 p.m.). Food and water were freely available. The animals were sacrificed by decapitation and the brain was quickly removed at 0–4 °C. Fresh tissue was prepared according to atlassections between Bregma 0.7 and 2.2 mm for nucleus accumebens (NAc). The experiments were approved by the Ethical Committee of the Pharmacology and Toxicology Section, Department of Pharmacy, following the European legislation (2010/63/EU) and were approved by Italian legislation on animal experimentation (Protocol No. 124/2003-A). Every effort was made to minimize animal suffering and to use the minimum number of animals necessary to obtain reliable results.

### 2.2. Preparation of Aβ Solutions

Synthetic human Aβ1-40 was dissolved in a CSF at a concentration of 100 M (stock solution). Then, this solution was filtered through a Millipore membrane with 0.2 µm pores and stored in small aliquots at −80 °C. Working solutions were freshly prepared by diluting an aliquot of the Aβ1-40 stock solution to the final concentrations used for in vitro analysis immediately prior to administration. Regarding the properties of the Aβ peptides that we used in our study, we previously showed that under our experimental conditions, the solutions of Aβ were mainly formed from Aβ monomers. However, we could not completely rule out the presence of small amounts of Aβ oligomers, since the aggregation is a concentration- and time-dependent process [23].

### 2.3. Release Experiments from Synaptosomes

Crude synaptosomes from rat NAc or hippocampus were prepared according to [24]. In release experiments, NAc synaptosomes were incubated for 20 min at 37 °C with [^3^H] Dopamine ([^3^H] DA; final concentration 0.03 µM) in the presence of 6-nitroquipazine (final concentration 0.1 µM), to avoid false labeling of serotonergic terminals and desipramine (final concentration 0.1 µM) and mislabeling of noradrenergic terminals. In a series of experiments, hippocampal synaptosomes were incubated with [^3^H] Noradrenaline ([^3^H] NA; final concentration 0.03 µM) in the presence of 6-nitroquipazine. Identical portions of the synaptosomal suspension were then layered onto microporous filters at the bottom of parallel superfusion chambers set at 37 °C [25,26]. Synaptosomes were superfused with standard physiological medium at 0.5 mL/min. Starting from t = 36 min to t = 48 min of superfusion, four consecutive 3 min fractions (b1–b4) were collected. Synaptosomes were exposed to agonists or to depolarizing agent (4-aminopyridine) at t = 39 min, until the end of superfusion, while other drugs (antagonists, peptides, antibody, allosteric modulators) were added 8 min before agonists. To introduce a specific amount of KLVFF into synaptosomes, we used an experimental approach previously used [27,28]. When indicated, rat NAc was homogenized in buffered sucrose containing Aβ1-40 (2 µM, 200 nM, 20 nM, and 2 nM) or 6E10 monoclonal antibody (1 mM) in order to entrap these agents into subsequently isolated synaptosomes. Based on estimates made by entrapping of [^3^H] sucrose, the intrasynaptosomal concentration of the compounds was approximately 5% of the original concentration in the homogenization medium [27]. The collected samples and superfused synaptosomes were then counted for radioactivity (fractional efflux). The agonist-induced effect was expressed as % induced overflow and evaluated by subtracting the level of neurotransmitter released in the four fractions collected under baseline conditions (no added drug) from the level released in the presence of the stimulus.

### 2.4. Flow Cytometric Analysis (FACS)

Crude synaptosomes from rat hippocampus were prepared according to the protocol. Consequently, 100 µL aliquots were separated to label the synaptosomes. Viability after 1 h incubation with KLVFF was assessed using a flow cytometer. Double staining allowed differentiation of early apoptosis and viable neuronal particles. Annexin-V (Miltenyi Biotec GmbH, Bergisch Gladbach, Germany; conjugate PE, order n° 130-118-363) has a high specificity and affinity for phosphatidylserine (PS); binding of Annexin-V to the membrane with exposed PS provides a highly sensitive method for detecting early cellular apoptosis [29,30]. Moreover, the viability was described by Calcein-AM (Biotium, Fremont, CA, USA; catalog n° 30026); this acetoxymethyl ester of calcein is a lipid-soluble diester fluorogenic esterase substrate that passively crosses the cell membrane and that is frequently used to stain viable cells. Within cells, it is converted by intracellular esterases into a polar lipid-insoluble fluorescent product (calcein) that is retained by particles with intact membranes but released from damaged neuronal buttons [31]. All assays were performed by Guava^®^ easyCyte™ Luminex Corporation Austin, TX, USA. Before incubation with Annexin-V, the sample was resuspended in 95 μL Annexin-V Binding Buffer. The synaptosome preparation was then enriched with 5 μL of Annexin-V conjugate, incubated for 15 min in the dark at room temperature, and finally washed again with binding buffer. Meanwhile, Calcein-AM working-solution was prepared, final concentration—2 μM (2 mM Calcein-AM stock solution diluted in PBS). An amount of 40 μL of the working solution was added to the sample and incubated at 37 °C for 30 min, then washed with MP. Some samples were labeled with both Annexin-V and Calcein-AM, to describe the ratio between apoptotic and non-apoptotic particles. In the latest phases of setting, Propidium iodide (PI) (Miltenyi Biotec, order n° 130-093-233) was added as a membrane integrity marker. It is excluded from viable vesicles but can penetrate membranes of dead or dying cells.

### 2.5. Statistical Analysis

Data are provided as mean ± SEM. Student’s *t*-test was applied to compare two independent groups. ANOVA with Tukey’s post hoc test was performed to compare more than two groups. For all tests, *p* < 0.05 was considered significant.

### 2.6. Chemicals

[7,8-3H] Dopamine (21.2 Ci/mmol (0.784 TBq/mmol)) and [1-7,8-3H] Noradrenaline (12.1 Ci/mmol (0.448 TBq/mmol)) were purchased from Perkin Elmer, Monza, Italy. Nicotine hydrogen tartrate salt was from Sigma (Sigma-Aldrich, St. Louis, MO, USA). 5-iodo-A-85380, α-conotoxin MII, oxotremorine, desformylflustrabromine hydrochloride, beta-amyloid (1–40) were from Tocris (Tocris Bioscience, Bristol, UK). AnnexinV conjugate PE was from Miltenyi Biotec GmbH (order n° 130-118-363) and CalceinAM was from Biotium (catalog n° 30026). KLVFF (segment Aβ 16-20) and VFLKF peptide were purchased from Aurogene s.r.l. (Rome, Italy)

## 3. Results

### 3.1. Flow Cytometry of Striatal Synaptosomes Exposed to KLVFF

**Figure 1:**
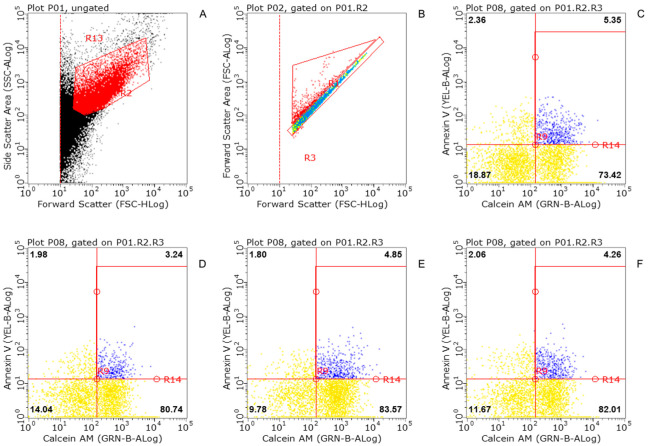
Flow cytometric analysis of striatal synaptosomes exposed to in or out KLVFF, or scramble peptide (VFLKF). (**A**) Representative density plot of synaptosomal preparation. (**B**) Singolet distribution of synaptosomal particles. (**C**) Calcein AM fluorescence versus Annexin V fluorescence for control synaptosomal particles. (**D**) Calcein AM fluorescence versus Annexin V fluorescence for KLVFF-treated synaptosomes (1 h). (**E**) Calcein AM fluorescence versus Annexin V fluorescence for VFLKF-treated synaptosomes (1 h). (**F**) Calcein AM fluorescence versus Annexin V fluorescence for KLVFF-entrapped synaptosomes. The percentage of total particles is shown for each quadrant. Particles positive for both markers are in the upper right quadrant.

The first step was to determine whether KLVFF causes damage to synaptosomes when administered directly at the concentration used to verify its modulating effect on membrane receptors. For this reason, we started cytofluorometric analysis of brain terminals (Figure 1A,B) preloaded with Calcein and Annexin V (Figure 1C). According to data in the literature [29,32,33], this experimental protocol can define viable and apoptotic particles. Interestingly, the exposure of synaptosomes for 1 h to 100 nM KLVFF does not increase the percentage of apoptotic signaling, describing the lack of apparent toxic effects (Figure 1D). Under the same conditions, we documented the absence of apoptotic signs also in the presence of scramble peptide VFLKF (Figure 1E). We had previously demonstrated that amyloid could interfere with synaptic receptors also binding cytoplasmatic targets [19]. Therefore, we decided to enrich inside synaptosomes with KLVFF taking advantage of the entrapping technique. Also in this configuration, KLVFF does not modify the viability of the synaptosomes (Figure 1F). The same protocol was used in the hippocampus to investigate different synapses. Figure S1 confirmed the absence of direct synaptotoxicity.

### 3.2. KLVFF-Mimics Aβ1-40 in the Regulation of nAChRs Modulating Dopamine Release

**Figure 2:**
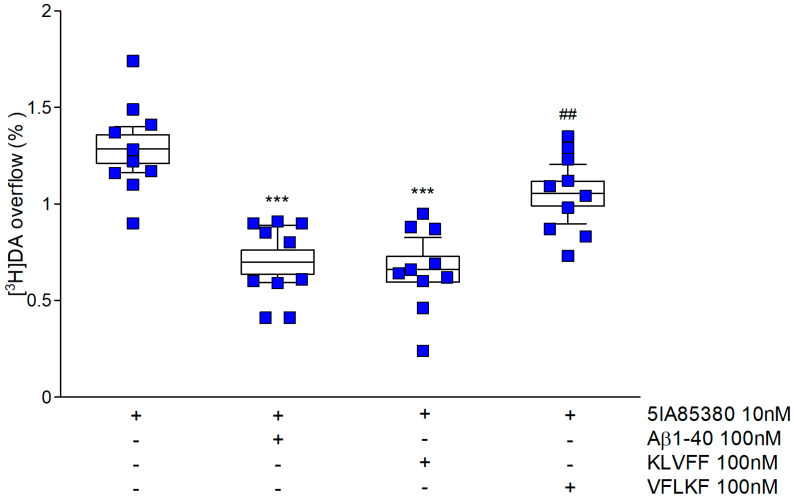
KLVFF counteracts the 5IA85380-induced [^3^H] dopamine overflow from striatal synaptosomes: comparison with full-length peptide and scramble fragment. Data are mean ± SEM (*n* = 6 per group). *** *p* < 0.001 (compared to 5IA85380); ## *p* < 0.01 (compared to 5IA85380 plus KLVFF) one-way ANOVA followed by Tukey’s post hoc test.

**Figure 3:**
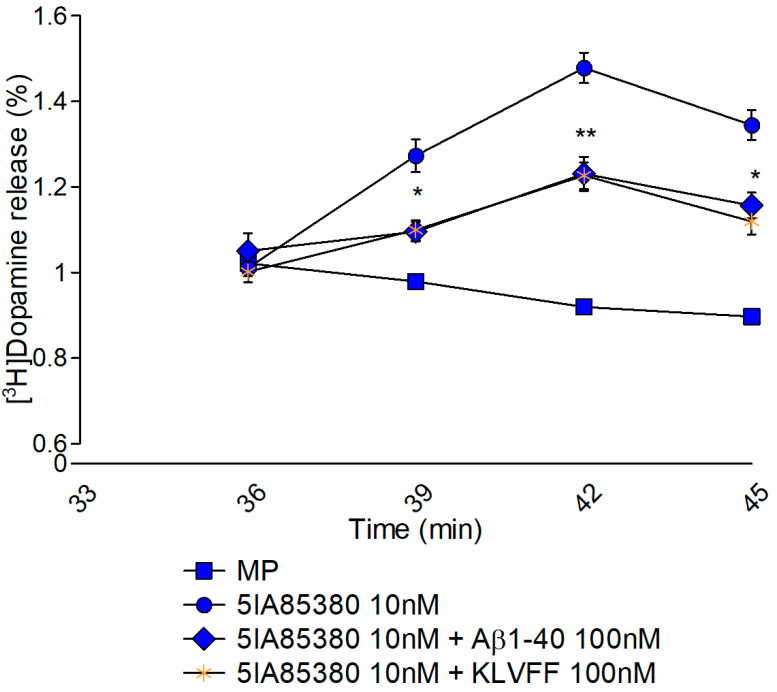
Time course of 5IA85380-induced [^3^H] dopamine release from striatal synaptosomes in the presence or in the absence of amyloid peptides. The results represent the fractional release obtained in the superfusion protocol. Data are mean ± SEM (*n* = 6 per group). * *p* < 0.05 ** *p* < 0.01 (compared to MP) two-way ANOVA followed by Tukey’s post hoc test.

Nicotinic receptors *(nAChRs)* regulating dopamine release in striatal synaptosomes are sensitive to 5IA85380, a selective α4β2 and α6β2 agonist (Figure 2). As previously demonstrated, 100 nM Aβ1-40 reduces the evoked dopamine overflow when administered to the nerve terminals during the perfusion phase [19]. Interestingly, the anti-aggregative peptide KLVFF mimics the full-length peptide, suggesting that the aminoacidic sequence 16–20 is crucial for the modulation of nAChRs. Accordingly, Figure 3 shows that the time courses of KLVFF and Aβ1-40 are almost overlapped. Consistent with the specificity of this mechanism, we found that VFLKF, a scramble, is completely ineffective under the identical experimental conditions.

### 3.3. Subtype-Selective Modulation of Nicotinic-Induced Dopamine Release

**Figure 4:**
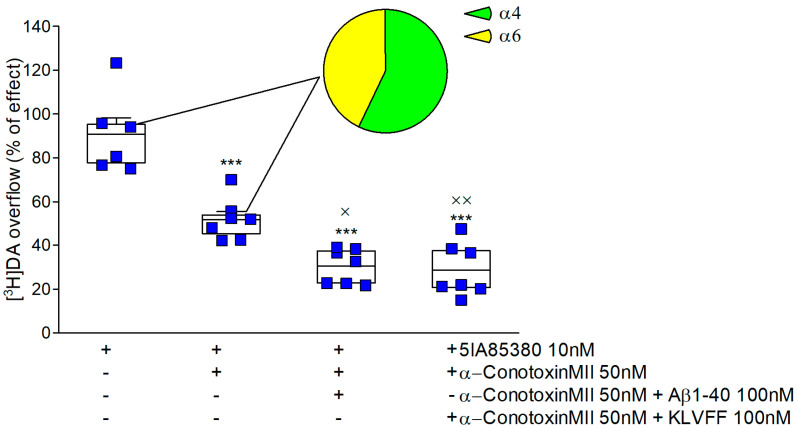
Effect of amyloid peptides on 5IA85380-induced [^3^H] dopamine release from rat NAc synaptosomes in the presence or in the absence of α-conotoxin MII. Data are mean ± SEM (*n* = 7 per group). *** *p* < 0.001 (compared to 5IA85380); × *p* < 0.05, ×× *p* < 0.01 (compared to 5IA85380 plus α-conotoxin MII) one-way ANOVA followed by Tukey’s post hoc test.

Since nAChRs inducing dopamine release can be distinguished into α-conotoxin MII sensitive and insensitive ones [34,35], we decided to investigate the effect of KLVFF on these different subtypes. Figure 4 shows that 5IA85380-induced overflow is significantly counteracted by α-conotoxin MII administration. These results confirm the presence of α6 containing nicotinic subtypes at the striatal nerve endings. Furthermore, the co-administration of Aβ1-40 or KLVFF does not alter α-conotoxin MII inhibition, suggesting that the peptides are also active in this subtype population.

### 3.4. KLVFF Effects on nAChRs Modulating Noradrenaline Release from Hippocampal Nerve Terminals

**Figure 5:**
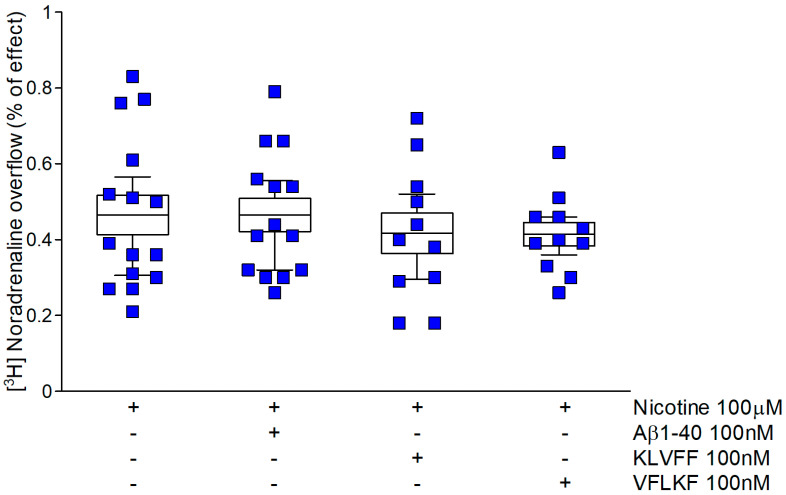
KLVFF is ineffective on the nicotine-induced [^3^H] noradrenaline overflow from hippocampal synaptosomes: comparison with full-length peptide and scramble fragment. Data are mean ± SEM (*n* = 12–15 per group).

It is well known that noradrenergic terminals express α3β4 subtypes, a group of presynaptic receptors particularly distinct from those active on dopaminergic neurons [36]. The specificity of amyloid regulation of nAChR function is confirmed examining norepinephrine release from hippocampal synaptosomes. Figure 5 shows that the nicotine-evoked noradrenaline overflow is completely insensitive to both Aβ1-40 and KLVFF.

### 3.5. Effects of Entrapped KLVFF on Nicotinic and Muscarinic Receptors

**Figure 6:**
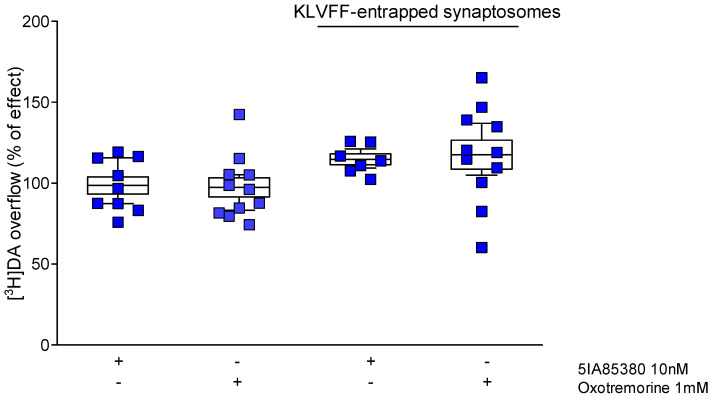
Effect of entrapped KLVFF on the nicotinic and muscarinic modulation of [^3^H] dopamine overflow from striatal nerve endings. Data are mean ± SEM (*n* = 8–12 per group).

In a previous work [19], we demonstrated that Aβ1-40 antagonizes both nicotinic and muscarinic receptors on dopaminergic terminals. In particular, using the entrapping protocol, we found that Aβ must cross synaptosomes to regulate muscarinic receptors. Otherwise, nicotinic receptors are insensitive to amyloid when treated with the entrapped peptide, suggesting the presence of an external binding site in their structure. Accordingly, entrapped KLVFF does not block nAChRs (Figure 6). Curiously, KLVFF is also inactive at muscarinic receptors when introduced into the cytosol. This finding seems to indicate that the amyloid active sequence on metabotropic cholinergic receptors is different from that affecting nAChRs.

### 3.6. Desformylflutrabromine Counteracts KLVFF on Dopaminergic nAChRs

**Figure 7:**
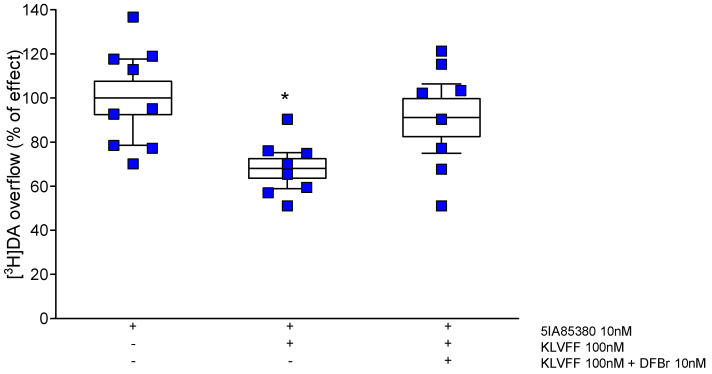
Effect of DFBr on the KLVFF modulation of 5IA85380-induced [^3^H] dopamine overflow from striatal synaptosomes. Data are mean ± SEM (*n* = 9 per group). * *p* < 0.05, (compared to 5IA85380) one-way ANOVA followed by Tukey’s post hoc test.

The negative modulation of nAChRs by KLVFF requires a full characterization and additional experiments are fundamental. Although, the possibility that KLVFF is a negative allosteric modulator on α4β2 subtypes is supported by the results shown in Figure 7. In fact, desformylflutrabromine (DFBr), a positive allosteric modulator of this nicotinic subtype, completely antagonizes the activity of KLVFF.

## 4. Discussion

Alzheimer’s disease is a terrible pathology without meaningful care [37]. In addition, the drug research process has failed in recent years on this issue. As if that were not enough, the last drug to be approved, Aducanumab, opens a great deal of debate about its effectiveness and safety [3,4]. Overall, despite great effort, the steps taken are not sufficient and any new therapeutic strategies are welcome. Accordingly, the possibility of intervening in the pathological aggregation of beta-amyloid is a compelling opportunity [9,10,14,15,38,39]. Starting with this project, we need to keep in mind that amyloid is an endogenous mediator in the healthy brain [7,40,41,42]. Strong evidence has extensively demonstrated that amyloid is important in LTP and memory consolidation [43,44,45]. The second point is that amyloid appears to be a wide-spectrum modulator, interfering with a plethora of targets [19,46,47,48,49,50,51]. Therefore, the ability to block only the derangement of this protein could be relevant to the success of the amyloid-based strategy. Different compounds can interfere with amyloid aggregation and, in particular, promising results have been obtained using amyloid fragments [16,17,18].

The KLVFF-based prototypes have been extensively discussed in the literature [16,17,18]. The scientific community agrees on the anti-aggregative properties of this small fragment [52,53,54]. At present, the possibility that the therapeutic use of this peptide will exhibit unexpected effects and primary side effects remains a stealth zone. Intriguingly, amyloid peptides, after integration with the membranes, promote the formation of calcium-permeable channels [55]. The first hypothesis that we formulated describes the possibility that KLVFF produces synaptotoxicity when administered to synaptosomes. Our cytofluorimetric analysis of synaptosomes showed that an amyloid fragment did not cause loss of viability when administered to the synaptosomes for 1 h. Supplementary results also confirmed safety when administered to hippocampal nerve endings.

Second, we speculated that KLVFF might maintain amyloid’s ability to modulate ionotropic or metabotropic receptors. Consistent with this hypothesis, our group gained good experience describing Aβ1-40 synaptic activities. A few years ago, using the entrapping technique during synaptosome formation, we demonstrated that amyloid has a different site of action at dopaminergic synapses [19]. We confirmed that amyloid has a specific affinity for the cholinergic targets. Nicotinic and muscarinic receptors, which regulate the release of dopamine, are inhibited by this peptide. Synaptosomes are an ideal model to study the functional interplay between amyloid and native targets [56]. Furthermore, by delivering the peptide directly into the cytoplasmic space, we found that external binding is essential to modulate nicotinic receptors. Conversely, the intracellular modulation of muscarinic receptors depends on an intracellular action of Aβ [6]. The lack of biophase due to the continuous perfusion of synaptosomes during the functional experiments ruled out the possibility that amyloid-peptides crossed the membrane to act on the muscarinic external binding site. Then, we decided to probe KLVFF in the same way to identify potential synaptic targets. The analysis revealed that KLVFF mimicked the full-length peptide and reduced dopamine release induced by the nicotinic agonist 5IA85380. This is surprising since little is known about the active sequence of amyloid on nicotinic receptors. This is true even though we have studied nicotinic receptors and amyloid compounds for many years. Interestingly, Pym and collaborators found that fragment 25–35 did not mimic all the effects of the full-length peptide on recombinant nicotinic subtypes [57].

The possibility of non-specific modulation is excluded by the absence of an effect when using the scrambled peptide. The time course of KLVFF closely resembles the modulation that occurred when we studied Aβ1-40. Nicotinic receptors that modulate dopamine release at the striatal level are ligand ion channels divided into different subtypes [36]. McIntosh revolutionized the characterization of these subtypes a few years ago by introducing the use of α-conotoxin MII [35]. This antagonist distinguishes two different groups of nicotinic receptors based on the presence of the α6 subunit. According to the literature, KLVFF mainly regulates α4-containing receptors. Furthermore, we can speculate that KLVFF modulates nicotinic receptors in an allosteric-reversible manner since a Positive Allosteric Modulator (PAM) such as DFBr is able to reverse the inhibition caused by the fragment. Several endogenous ligands have been reported to have negative allosteric influences on nAChRs such as anandamide, progesterone, neurosteroids, and proteins such as Lynx-1 [58]. The selectivity of KLVFF modulation for specific nicotinic receptor subtypes is confirmed by our data on hippocampal noradrenergic synaptosomes. Accordingly, α3β4 nicotinic receptors are expressed at these nerve terminals. KLVFF is ineffective on these subtypes, confirming the lack of effects of the full-length protein. Accordingly, data in the literature describe different allosteric modulators for α4β2 and α3β4 subtypes [58]. Hippocampal norepinephrine release may be a fundamental target in the memory impairment that characterizes AD [59]. In addition, norepinephrine release is involved in the inflammatory cascade that occurs in several neurodegenerative diseases [60,61]. Our data indicate that nicotinic receptors at noradrenergic terminals in the hippocampus are not a functional target for the monomeric peptides. Additional studies are needed to investigate the role of noradrenergic transmission in AD and the effects of amyloid. Interestingly, KLVFF entrapped in the cytoplasmic space of synaptosomes failed to modulate either nicotinic or muscarinic receptors, which stimulated dopamine release. This is a fundamental step in describing the selectivity of amyloid at cholinergic receptors. In fact, we have previously shown that Aβ1-40 modulates muscarinic receptors (probably M5) under the same conditions. Figure 6 seems to indicate that the active sequence of amyloid on muscarinic receptors may differ from that inhibiting nicotinic receptors. To the best of our knowledge, this is the first time that functional data have suggested distinct active amyloid domains affecting the cholinergic receptors. Obviously, this is an indication and further experiments are essential to describe this feature.

## 5. Conclusions

The KLVFF fragment possesses modulatory functions at nicotinic receptors located on dopaminergic terminals. Furthermore, we highlighted the absence of direct synaptotoxicity of this compound by describing the lack of apoptotic damage during a relatively prolonged exposure of synaptosomes at nanomolar concentrations. This research is a first step towards a deeper understanding of the potential interactions of this promising anti-aggregative peptide to reduce side effects in AD patients.

## Data Availability

All original data are available upon request.

## Supplementary Materials

The following supporting information can be downloaded at: https://www.mdpi.com/article/10.3390/biomedicines10092231/s1, Figure S1: Flow cytometric analysis of hippocampal synaptosomes exposed to KLVFF.
