# Supplementary material for: The Anti-Aggregative Peptide KLVFF Mimics Aβ1-40 in the Modulation of Nicotinic Receptors: Implications for Peptide-Based Therapy
Source: Biomedicines. 2022 Sep 8;10(9):2231. doi: 10.3390/biomedicines10092231 (PMC9496455; doi:10.3390/biomedicines10092231)
Supplement: Supplementary file 1 [file biomedicines-10-02231-s001.zip › biomedicines-1863379-SI.pdf]

biomedicines

MDPI

**Supplementary**

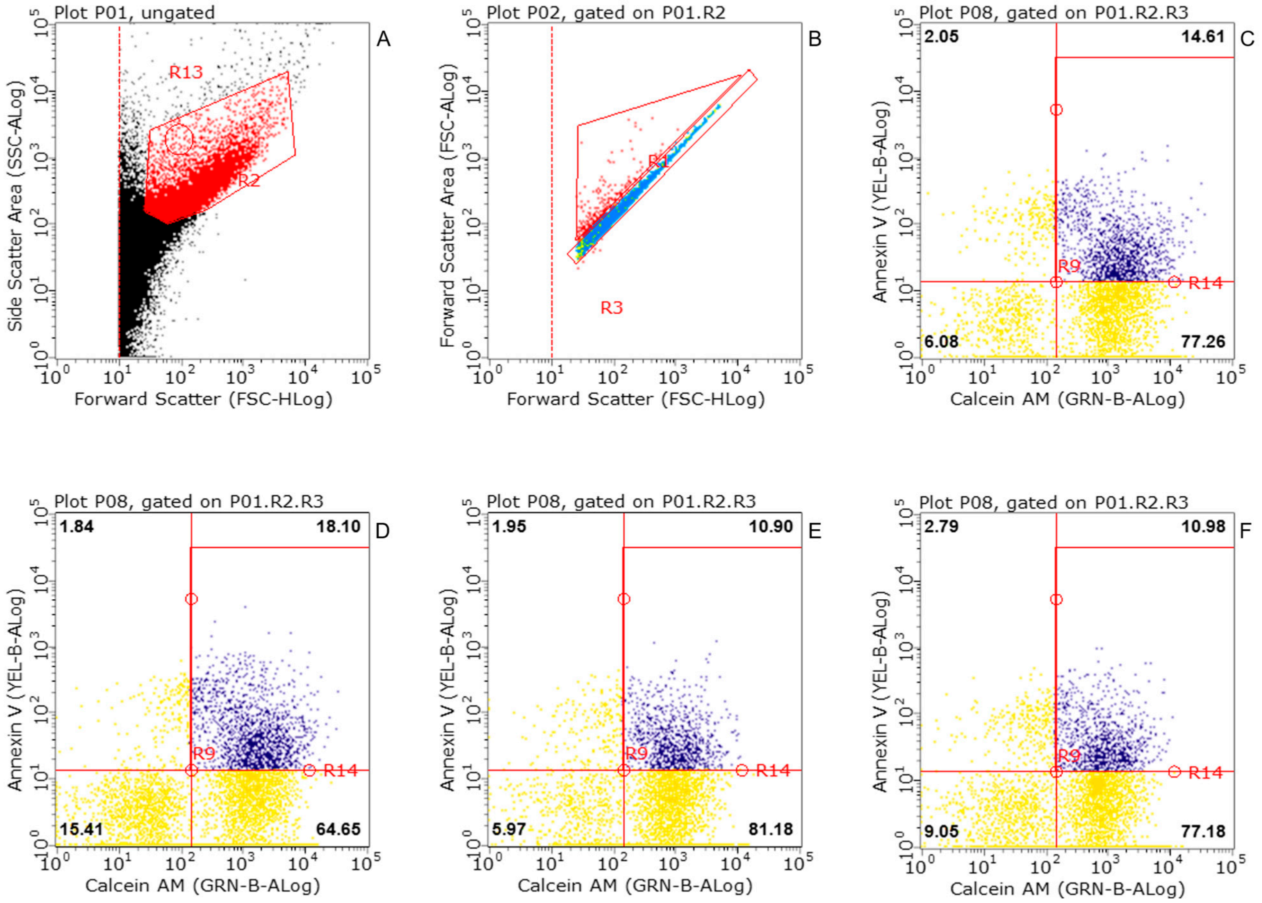


**Figure S1.** Flow cytometric analysis of hippocampal synaptosomes exposed to KLVFF. (A) Representative density plot of synaptosomal preparation. (B) Singolets distribution of synaptosomal particles. (C) Calcein AM fluorescence versus Annexin V fluorescence for control synaptosomal particles. (D) Calcein AM fluorescence versus Annexin V fluo-rescence for KLVFF-treated synaptosomes (1 hour). (E) Calcein AM fluorescence versus Annexin V fluorescence for VFLKF-treated synaptosomes (1 hour). (E) Calcein AM fluorescence versus Annexin V fluorescence for KLVFF-entrapped synaptosomes. The percentage of total particles is shown for each quadrant. Particles positive for both markers are in the upperright quadrant.
